# Increasing Coverage of Proteome Identification of the Fruiting Body of *Agaricus bisporus* by Shotgun Proteomics

**DOI:** 10.3390/foods9050632

**Published:** 2020-05-14

**Authors:** Tae-Ho Ham, Yoonjung Lee, Soon-Wook Kwon, Myoung-Jun Jang, Youn-Jin Park, Joohyun Lee

**Affiliations:** 1 Department of Crop Science, Konkuk University, Seoul 05029, Korea; lion78@daum.net (T.-H.H.); yoon10.lee@gmail.com (Y.L.); 2 Department of Crop Plant Bioscience, Pusan National University, Milyang 50463, Korea; swkwon@pusan.ac.kr; 3 Department of Plant Resources, Kongju National University, Yesan 32439, Korea; plant119@kongju.ac.kr; 4 Kongju National University Legumes Green Manure Resource Center, Yesan 32439, Korea; cocono@naver.com

**Keywords:** *Agaricus bisporus*, shotgun proteomics, gene ontology

## Abstract

To increase coverage of protein identification of an *Agaricus bisporus* fruiting body, we analyzed the crude protein fraction of the fruiting body by using a shotgun proteomics approach where 7 MudPIT (Multi-Protein identification Technology) runs were conducted and the MS/MS spectra from the 7 MudPIT runs were merged. Overall, 3093 non-redundant proteins were identified to support the expression of those genes annotated in the genome database of *Agaricus bisporus*. The physicochemical properties of the identified proteins, i.e., wide pI value range and molecular mass range, were indicative of unbiased protein identification. The relative quantification of the identified proteins revealed that K5XI50 (Aldedh domain-containing protein) and K5XEW1 (Ubiquitin-like domain-containing protein) were highly abundant in the fruiting body. Based on the information in the Uniprot (Universal Protein Resource) database for *A. bisporus*, only approximately 53% of the 3093 identified proteins have been functionally described and approximately 47% of the proteins remain uncharacterized. Gene Ontology analysis revealed that the majority of proteins were annotated with a biological process, and proteins associated with coiled-coil (12.8%) and nucleotide binding (8.21%) categories were dominant. The Kyoto Encyclopedia of Genes and Genome analysis revealed that proteins involved in biosynthesis of secondary metabolites and tyrosine metabolism were enriched in a fruiting body of *Agaricus bisporus*, suggesting that the proteins are associated with antioxidant metabolites.

## 1. Introduction

The button mushroom *Agaricus bisporus*, a species of macrofungi of the phylum Basidiomycota, is a typical edible fungus, together with the oyster mushroom. Since the start of *A. bisporus* cultivation in the 1650s, several methods of cultivation have been developed [1]. Currently, this mushroom is widely cultivated and consumed globally, particularly in the Netherlands and USA. In Korea, the cultivation of *A. bisporus* was introduced in the 1950s. At present, this mushroom accounts for approximately 7% of the mushroom cultivation industry in Korea, with an annual output ranged 6678 to 13,052 tons in last 10 years [2].

Sustainable development of the mushroom industry in Korea requires breeding new cultivars with superior traits that meet the demands of producers and consumers. Genetic studies of the traits that are fundamental for breeding programs, however, reveal a suppressed recombination frequency of co-segregating markers, resulting in limited and unsaturated genetic linkage maps [3]. Recent genomic methods constitute a possible alternative approach for gaining useful genetic information for the *A. bisporus* breeding program. The haploid genome sequence of *A. bisporus* is available; the genome is comprised by 13 chromosomes and is 30.4 Mb in size [4]. This information has enabled various studies of the genome structure and gene expression of *A. bisporus*.

Proteomics is a powerful tool used for the detection and identification of gene expression and constitution, respectively [5]. With the advances in mass spectrometry technology, proteomics is becoming increasingly relevant for the study of fungal biology, especially the physiology and development of filamentous fungi [5]. As research into *A. bisporus* has started to attract attention, proteome studies have also advanced from the determinations of amino acid composition [6] to two-dimensional polyacrylamide gel electrophoresis (2-D PAGE) [7,8]. Since its original publication, the O’Farrell method for two-dimensional electrophoresis of proteins has been widely used in proteomics [9]. The advantages of this method are that it is easy to apply, and the data are obtained within a short timeframe. However, 2-D PAGE is not suitable for the identification of low-abundance proteins; high-molecular weight or small-molecular weight proteins; hydrophobic proteins; or proteins with a basic pI value, which is the pH at which the net charge of the protein is zero [10]. To overcome the limitations of 2D-PAGE, relevant shotgun proteomics methods recently emerged [11]. Shotgun proteomics, also known as MudPIT, allows for large-scale identification of proteins, which is an area where 2D-PAGE does not perform well [12]. At the same time, shotgun proteomics takes a lot less time than 2D-PAGE analysis [13]. 

Previous studies of the button mushroom have mainly focused on its cultivation or breeding [14]. Shotgun proteomics in crops has only been used to analyze a few model fungi because this method relies on an available complete genome. In the current study, to provide basic -omics information for *A. bisporus*, we inferred the proteome of a fruiting body of the species *A. bisporus* using shotgun proteomics approaches though merging 7 MudPIT runs.

## 2. Materials and Methods

### 2.1. Cultivation of A. bisporus

The cultivar of Sae-han (strain ASI1350), which was obtained from Gyeongsangbuk-do Agricultural Research and Extension Services in Korea (http://www.gba.go.kr/) was prepared as a grain spawn. Mushrooms were grown in a plastic bag filled with 2 kg of the button mushroom compost. The compost was prepared from wheat straw (65% total dry weight), poultry manure (28%), gypsum (4%), limestone (2%), and urea (1%) at pH 8.1 and with 68% moisture. The grain spawn was used to inoculate the compost at 1% concentration. This was followed by a 20 d incubation at 22–23 °C and 60–70% relative humidity in darkness. After the spawn-run, the compost was colonized by 80–90% mycelium of *A. bisporus* and then covered with a 3–4 cm deep layer of the casing soil. The temperature in the cultivation room was adjusted to 17 °C, and the relative humidity was maintained at approximately 90%. The room was ventilated to induce fruiting body formation when the mycelium reached the surface of the casing layer under 14 h light and 10 h dark conditions.

### 2.2. Protein Extraction

For 7 MudPIT runs, the harvested 7 fruiting bodies (cap) of *A. bisporus* were stored at –80 °C, powdered in liquid nitrogen, and placed in a 1.5 mL Eppendorf tube. Extraction buffer (8 M urea, 5 mM dithiothreitol, 1% LDS, and 100 mM Tris, pH 8.5) was then added, and the powdered samples were homogenized in the buffer. The homogenized samples were centrifuged at 14,000× *g* for 15 min at 4 °C, and the supernatant was transferred to a new tube. The supernatant was then filtered through membrane filters (0.45 µm size), and the protein was precipitated overnight in the presence of 20% (*v*/*v*) trichloroacetic acid. The pellet was washed several times with cold acetone to remove pigments. The protein was then resolubilized in a resolubilization buffer (8 M urea and Tris-HCl, pH 8.5). The concentration of the protein was determined by using 2D-protein Quant kit (GE Healthcare, Piscataway, NJ, USA) as described elsewhere [15].

### 2.3. Protein Digestion

For the experiment, 300 μg of the sample protein was reduced using Tris (2-carboxyethyl) phosphine hydrochloride (TCEP), by adjusting the concentration of TCEP to 5 mM and incubating for 30 min at room temperature. The reduced sample was carbamidomethylated in the presence of 10 mM iodoacetamide during incubation for 30 min at room temperature in the dark. The protein sample was then diluted with 100 mM Tris-HCl to reduce the urea concentration to 2 M, and CaCl_2_ was added to a final concentration of 2 mM. Then, 5 μg trypsin and trypsin buffers were added. The sample was incubated overnight at 37 °C to allow for protein digestion. The digested proteins were desalted by passing through a SPEC PLUS PT C18 column (Agilent Technologies, Santa Clara, CA USA), and the solvent was evaporated by using a speed-Vac.

### 2.4. Liquid Chromatography–Tandem Mass Spectrometry (LC-MS/MS) Analysis

Nano LC connected to Finnigan LTQ mass spectrometer (Thermo Scientific, Waltham MA) was used. Biphasic columns were prepared in-house for analysis; the columns contained 365 μm OD (outer diameter) × 100 μm ID (inside diameter) fused-silica capillaries (Polymicro Technologies, Phoenix, AZ, USA). The capillaries were packed using a pressure cell with helium and 9 cm of C18-AQ 5 μm reverse phase (PP), followed by 3 cm of 5 μm strong cation exchange Luna resin. The desalted sample was loaded onto the in-house column in 20 μL of a mixture of 5% acetonitrile and 0.1% formic acid.

Reversed-phase chromatography was performed using a binary buffer system of 0.1% formic acid (buffer A) and acetonitrile in 0.1% formic acid (buffer B). Nano LC was performed by using a linear gradient of 3–50% of buffer B at a flow rate of 0.200 μL/min. The peptides were eluted in the course of an 11-step program of increasing concentration of salt solution. The eluent was ionized by electrospraying from the column directly into the MS/MS system. A parent-ion scan was performed in the range of 400–1600 m/*z* (mass-to-charge ratio). The top five most intense parent ions were chosen, and an MS/MS-ion scan was performed by collusion-induced dissociation. The run time for LC-MS/MS was 120 min for each step. The total run time for the 11 steps was approximately 22 h. A total of 7 MudPIT runs were conducted. 

### 2.5. Proteomic Data Analysis

The MS/MS spectral files for 7 MudPIT runs were merged in the single file. For each identified protein, protein ID, spectra count, pI value, and molecular mass were determined by using the Proteome Discoverer software (version 1.3) (Thermo Fisher Scientific, Waltham, MA, USA) with the merged single MS/MS spectra file. The fragmentation spectra data for *A. bisporus* were searched using Uniprot (http://www.uniprot.org/). For database searching, carbamidomethylation of cysteine was set as a fixed modification, and oxidation of methionine was set as a variable modification. To verify these modifications, protein identifications were filtered at 1% false discovery rate.

### 2.6. Determination of the Relative Protein Abundances

The output of the Proteome Discoverer analysis was exported to Microsoft Excel to calculate the normalized spectral counts (NSpC). The NSpC for each protein k is given by the equation

(1) $${\left(\mathrm{NSpC}\right)}_{k}=\frac{{\left(\frac{\mathrm{SpC}}{L}\right)}_{k}}{\sum_{i=1}^{n}{\left(\frac{\mathrm{SpC}}{L}\right)}_{i}}$$

where the total number of MS/MS spectra matching peptides from the protein k (SpC) is divided by the protein’s length (L) and then divided by the sum of all SpC/L values for the protein in the experiment.

### 2.7. Bioinformatics Analysis

Gene Ontology (GO) annotations for *A. bisporus* were from Uniprot (http://www.uniprot.org/). GO analysis and Kyoto Encyclopedia of Genes and Genome (KEGG) pathway analysis was performed using the DAVID (Database for Annotation, Visualization and Integrated Discovery, version 6.8) (https://david.ncifcrf.gov/) and the *A. bisporus* Uniprot database was used as a reference. Protein GO classification was performed by using PANTHER (Protein ANalysis THrough Evolutionary Relationships, version 14.0, Los Angeles, CA, USA) (http://www.pantherdb.org/) classification system and CateGOrizer (http://www.animalgenome.org/tools/catego/). The enriched function-related proteins were grouped. Transmembrane domains were predicted using TMHMM (version 2.0, Lyngby, Denmark) (http://www.cbs.dtu.dk/services/TMHMM/) with protein sequence data from Uniprot.

## 3. Results and Discussion

### 3.1. Identified Proteins of the A. bisporus Fruiting Body

Overall, the shotgun proteomic analysis of the fruiting body of the species *A. bisporus* identified 3093 proteins (Supplementary Table S1). Compared with the 2D-PAGE technology that can generally resolve approximately 1000 protein spots in a single gel, the shotgun proteomic analysis conducted in the current study had a much higher resolving power, identifying ~1000 proteins for a single MudPIT run. A phenomenon of analytical incompleteness in MudPIT analysis was reported previously [16], in which any single analytical run may only identify a fraction of the relevant peptides in a highly complex mixture of peptides. Therefore, for deep proteome identification, we conducted 7 MudPIT runs with the fruiting bodies of the Sae-han cultivar, and the MS/MS spectra were merged to a single file. From the protein identification with the single merged MS/MS file, we identified 3093 non-redundant proteins of the fruiting body of *A. bisporus*. After 5 MudPIT runs were merged, the number of newly identified proteins was less than 100 as 6, 7 MudPIT runs were merged. Thus, to increase the coverage of protein identification more than our results, other strategies are required such as protein sample fractionation or solubilizing hydrophobic proteins. In addition to increasing the protein identification coverage, the confidence level of the protein identification also increased. At least one unique peptide was assigned to the identified proteins. The spectral counts that represent the number of MS/MS spectra assigned to a peptide from certain proteins were found to have increased the identified proteins. The minimum number of spectral counts for the identified protein was 81 for K5Y688 (CS domain-containing protein) and the maximum was 16,914 for K5XHR7 (uncharacterized protein). Around 80% of the identified proteins showed more than 1000 spectral counts. (Supplementary Table S1). Spectral counting has become a commonly used approach for measuring relative abundance of proteins in label-free shotgun proteomics [17]. The increased number of the spectral count for the identified pterions improved acquiring relative abundance of proteins.

**Figure 1:**
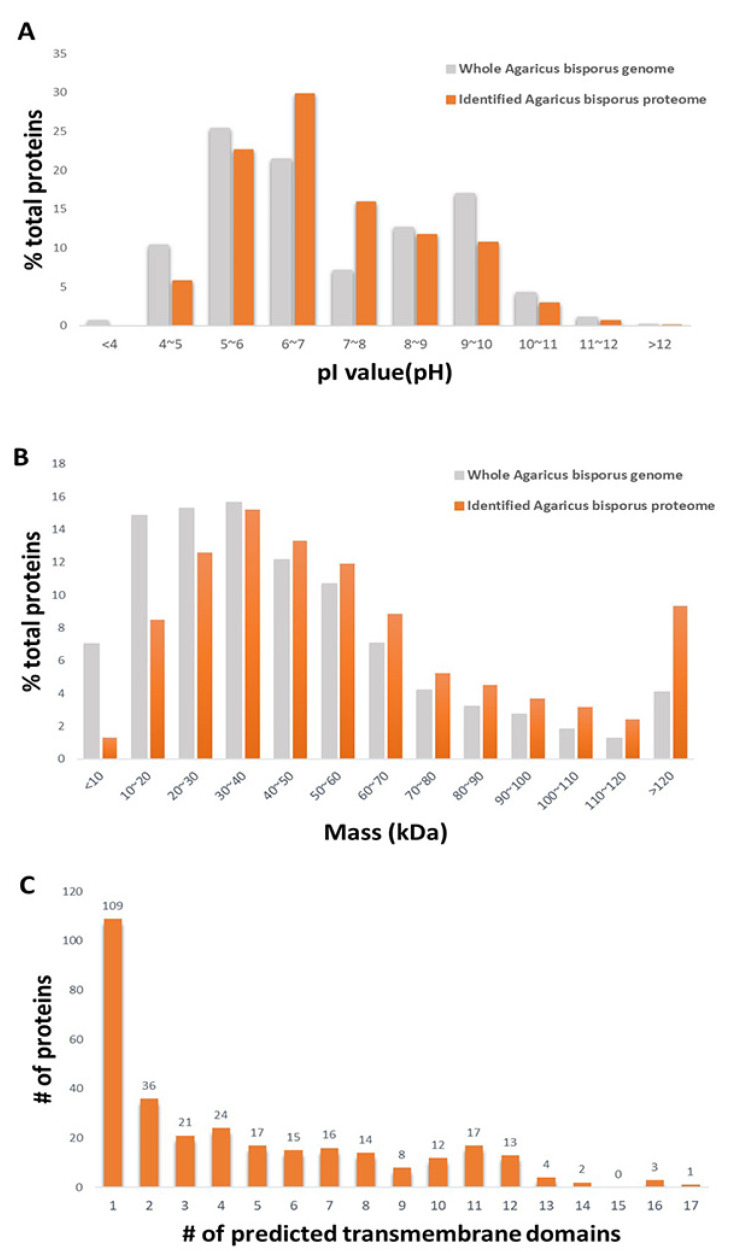
Bioinformatic analysis of the identified proteins. (**A**) Distributions of isoelectric point (pI). (**B**) Distributions of molecular mass. (**C**) Number of predicted transmembrane domains.

The distributions of the physiochemical properties of the identified proteins, pI values, and molecular masses were analyzed. The pI value and molecular mass are the main and limiting factors that affect protein resolution in 2D-PAGE because of the gel properties. The distributions of the pI values and molecular masses of the identified proteins were compared with those of proteins predicted from the whole *A. bisporus* genome sequence (Figure 1A,B). 

K5XUF0 (uncharacterized protein) had the highest pI value at 12.19. K5VUP2 (uncharacterized protein) had the lowest pI value at 4.01. Based on the distribution of the pI values of the identified proteins, approximately 42% of proteins had a pI higher than 7.0, which, in general, would limit their resolution on 2D-PAGE. No proteins with a pI value below 4 were detected, even though such proteins are putatively encoded by the *A. bisporus* genome. That is probably because of the low proportion of acidic proteins encoded by the fungal genome. The molecular mass of the identified proteins ranged from 5.6 kDa (K5XCV4; uncharacterized protein) to 547.6 kDa (K5VXU2; Midasin). Based on the molecular mass data, the mass distribution of the identified proteome was similar to that of the predicted whole proteome. However, the proportion of identified proteins with a molecular mass lower than 30 kDa was smaller than that predicted from the whole genome, and the proportion of proteins with a molecular mass higher than 120 kDa was higher than that predicted from the whole genome. The distribution of the identified proteins showing a wide range of molecular weight and isoelectric points suggested that there was little bias or information loss with the methods used in deep proteome identification of the fruiting body of the species *A. bisporus*. Protein having transmembrane domains predicted by Transmembrane Helices Hidden Markov Models (TMHMM) were explored (Figure 1C, Supplementary Table S2). Based on our protein extraction and solubilization methods, the extraction method was specific for soluble proteins. Even though neither cellular organelles nor cellular membrane were purified in this experiment, 312 proteins were predicted to have transmembrane domain. Interestingly, the functions of all of the 312 proteins were unknown. This result represents insufficient database accumulation for the gene functions encoded in the genome of *A. bisporus.* Based on the fact that the prediction programs used is not perfect, some of the predicted proteins may not be membrane bounded proteins; however, this result suggests that some of the membrane bound proteins are possibly identified by the MudPIT analysis applied in our experiment without additional membrane fractionation procedure.

### 3.2. Highly Abundant Proteins in the Fruiting Body of A. bisporus

We next used the spectral counts method [18] to determine the relative abundance of the identified proteins. The method considers the spectral counts in the MS/MS spectra data, normalized as NSpC, to determine the relative protein abundance as a proportion of the sum of the relative abundances of all the identified proteins. The abundance of the identified proteins was compared with NSpC. Even though the statistical analyses were not applied in this comparison, the high score of NSpC represents its high abundance in the cell of the fruiting body. Unlike the plant leaf proteome, in which RubisCO and photosynthesis-associated proteins are highly dominant, no highly dominant proteins were apparent in the fruiting body of the species *A. bisporus*. The abundance of the top ten highly abundant proteins accounted for 3.0% of all proteins (Table 1). Among the 3093 identified proteins, the most abundant protein was K5XI50 (Aldedh domain-containing protein), which is the conserved domain of the Aldehyde Dehydrogenase (ALDH) superfamily, which is known to be involved in metabolism and abiotic/biotic stress responses [19]. K5XEW1 (Ubiquitin-like domain-containing protein) was another highly abundant protein in the fruiting body. This protein is found across the Eukarya and is involved in the regulation of signal transduction and enzymatic activity [20]. Ribosomal proteins such as ribosomal_S10 domain-containing protein and SBDS domain-containing protein were also abundant in the fruiting body. Interestingly, transcription elongation factor TEF EF1B localized in the nucleus was highly present.

### 3.3. Functional Classification and GO Analysis

**Figure 2:**
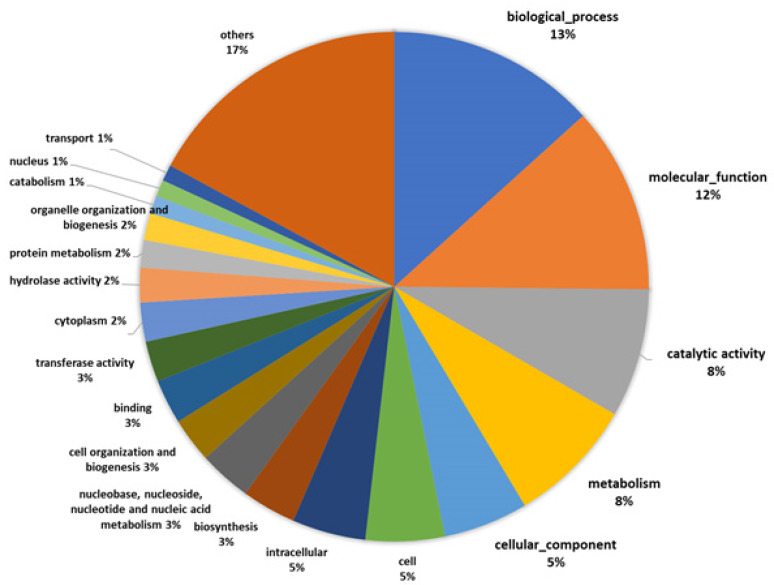
Gene Ontology (GO) functional categorization.

Based on the information for *A. bisporus* in the Uniprot database, we were able to determine a function for 53% of the 3093 identified proteins whereas for 47% we were unable to determine their functions. Among the top ten highly abundant proteins, only five were functionally described. This indicates that even though the completed genome sequence information is available, follow-up studies, such as functional annotation and gene expression, are still required. The detection of uncharacterized proteins in the fruiting body of the species *A. bisporus* in the current study supports their possible role and presence in the fruiting body. GO information was available for 2543 of the identified proteins. We used GO analysis to categorize the proteins into 89 groups according to their GO functions. The highest proportion of proteins represented the category biological process (13.3%), followed by molecular function (11.9%), catalytic activity (8.2%), metabolism (8.1%), and cellular component (5.4%) (Figure 2). 

**Figure 3:**
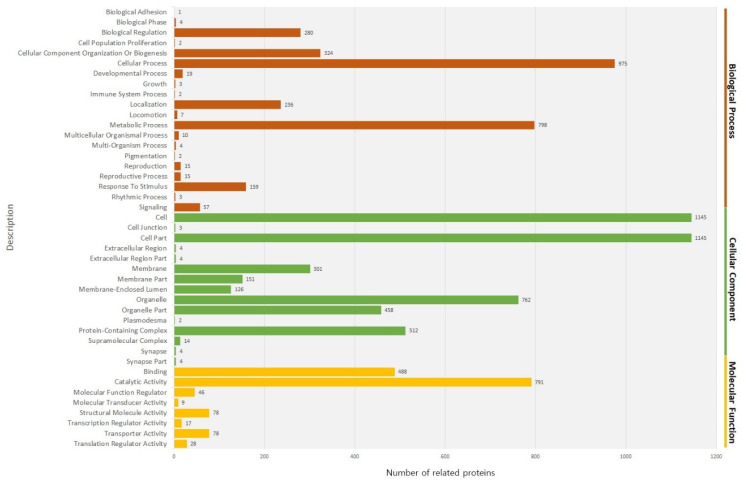
GO analysis describing the three main categories, biological process, cellular component, molecular function.

We then conducted GO enrichment analysis to compare the proportion of the GO terms for the predicted proteins encoded by the genome and the proteins identified in the fruiting body. In the proteins identified in the fruiting body, 20 GO terms for biological processes, 15 GO terms for cellular components, and 8 GO terms for molecular functions were recovered (Figure 3).

Among the GO terms for biological processes, the proportion of proteins associated with a cellular process and metabolic process was high. Among GO terms for the cellular components, the proportion of proteins associated with cell compartment and the cell was high. Among the proteins associated with molecular functions, the proportion of proteins associated with catalytic activity and binding was high. When functionally related proteins were grouped, 70 enriched groups were apparent (Supplementary Table S3). The proteins grouped in the coiled-coil (12.8%) and nucleotide binding (8.21%) categories were dominant. The coiled-coil proteins are thought to facilitate the expansion of the centrosome to aid cell division [21]. The enriched proteins involved in nucleotide binding and coiled-coil proteins may represent the cell division status of the fruiting body of the species *A. bisporus*. Further, the enrichment of proteins related to metabolic pathways and secondary metabolites is possibly associated with the variety of nutrients present in the fruiting body of the species *A. bisporus* [22]. To test how well our dataset covers known cellular pathways, the identified fruiting body proteome was overlaid onto the pathway database of the KEGG [23] (Table 2). 

KEGG pathway enrichment analysis was performed by using DAVID. There were 23 pathways obtained and 18 of them were statistically significant (*p* < 0.05). Among the 3093 proteins, only 23% (731 proteins) were assigned to a KEGG pathway. Of these, the category of metabolic pathway was highly enriched. The highly enriched metabolic pathway in the fruiting body of the species *Auricularia heimuer* has been previously reported in a gene expression study [24]. A total of 186 proteins were assigned to the category of secondary metabolites, and three of them were assigned to terpenoid backbone biosynthesis. Terpenoids are derived from the five-carbon isoprene units and consist of multicyclic structures that differ from one another on the basis of carbon skeleton and different functional groups [25]. Terpenoids isolated from mushrooms have been reported that are associated with various pharmacological activities such as anticancer, anticholinesterase, antiviral, antibacterial, anti-inflammatory, and antioxidase activities [26]. A total of 18 proteins were assigned to the category of tyrosine metabolism. Tyrosine, as a building block, is a precursor for phenolic compounds [27] which are effective antioxidants. Edible mushrooms as a food are sources of protein, fiber, vitamins, minerals, and useful bioactive metabolites with a broad spectrum of pharmacological benefits such as antioxidant activity [28]. Button mushrooms as a food source for an antioxidant can be used to help the organism to reduce oxidative damage [29]. The detected proteins associated in the pathway of antioxidant in the fruiting body of the species *A. bisporus* are possible candidate genes for developing high quality button mushroom cultivars as an antioxidant-containing food.

## 4. Conclusions

Using a shotgun proteomics approach where merging 7 MudPIT runs increased coverage of protein identification and the confidence level of the identified protein, we identified 3093 proteins in the fruiting body of the species *A. bisporus*. The physiochemical properties of the identified proteins (the pI values and molecular masses) indicated unbiased protein identification. Because of the lack of gene or protein expression data for *A. bisporus*, only approximately 53% of the identified proteins were functionally described and approximately 47% of the proteins are still uncharacterized. The current study is the first report presenting a list of over 3000 proteins in the fruiting body of the species *A. bisporus*. The shotgun proteomics approach and the complete genomic database allowed us to identify more than 2000 non-redundant proteins. Unfortunately, the function of approximately 47% of the identified proteins has not yet been described because genomic and annotation analyses of *A. bisporus* are limited, unlike in other model organisms. However, proteomic detection of these proteins may act as supporting evidence for their existence. The shotgun proteomic approach employed in the current study could be used in further studies comparing the proteome of the fungus *A. bisporus* grown in certain environments or at certain developmental stages.

## Figures and Tables

**Table 1:** Top 10 highly abundant proteins in the fruiting body of *Agaricus bisporus*.

Accession	Description	Score	# Proteins	# Peptides	# PSMs	# AAs	MW [kDa]	calc. pI	NSpC
K5XI50	Aldedh domain-containing protein	253.09	2	14	10,609	107	11.60162	5.046387	0.005653
K5XEW1	Ubiquitin-like domain-containing protein	323.58	3	59	10,190	159	18.03673	9.979004	0.003654
K5Y3E9	Uncharacterized protein	232.87	1	45	8737	161	18.49427	4.665527	0.003094
K5VUM2	Ribosomal_S10 domain-containing protein	157.35	1	55	6348	124	13.7115	9.671387	0.002919
K5XEM2	Uncharacterized protein	203.94	1	26	5533	112	11.29655	4.335449	0.002817
K5X 7C3	Uncharacterized protein	244.78	1	52	13,010	277	30.24033	5.160645	0.002678
K5X9Z8	SBDS domain-containing protein	212.38	1	29	5666	125	13.97901	6.303223	0.002585
K5Y5Z1	Uncharacterized protein	214.95	1	59	6529	147	16.15384	10.18408	0.002532
K5WMW0	Transcription elongation factor TEF EF1B	282.18	1	48	9256	212	23.10943	4.703613	0.002489
K5XAS4	Uncharacterized protein	313.32	1	76	8200	190	21.39238	9.495605	0.002461

Score: the sum of the ion scores of all peptides that were identified; coverage: the percentage of the protein sequence covered by identified peptide.; # proteins: the number of identified proteins in a protein group; # unique peptides: the number of peptide sequences that are unique to a protein group; # peptides: the total number of distinct peptide sequences identified in the protein group; # PSMs: the total number of identified peptide spectra matched for the protein; # AAs: the total number of amino acid in the protein; MW [kDa]: molecular masses of protein; pI: isoelectric points; NSpC: normalized spectral counts.

**Table 2:** KEGG Pathway analysis of identified *Agaricus bisporus* proteins.

KEGG Pathway Term	Number of Related Proteins	Percentage (%)	*p*-Value
2-Oxocarboxylic acid metabolism	27	1.85	1.70 × 10^−3^
Alanine, aspartate and glutamate metabolism	25	1.71	4.40 × 10^−4^
Arginine biosynthesis	14	0.96	1.70 × 10^−2^
Biosynthesis of amino acids	81	5.54	3.40 × 10^−8^
Biosynthesis of antibiotics	148	10.12	1.80 × 10^−5^
Biosynthesis of secondary metabolites	186	12.71	1.50 × 10^−3^
Carbon metabolism	79	5.40	4.80 × 10^−6^
Citrate cycle (TCA cycle)	24	1.64	5.50 × 10^−3^
Endocytosis	49	3.35	2.60 × 10^−2^
Glycolysis/Gluconeogenesis	36	2.46	2.00 × 10^−2^
Metabolic pathways	424	28.98	2.80 × 10^−2^
Methane metabolism	19	1.30	3.30 × 10^−2^
Propanoate metabolism	10	0.68	3.40 × 10^−2^
Proteasome	35	2.39	4.10 × 10^−6^
Ribosome	67	4.58	4.70 × 10^−3^
RNA transport	69	4.72	1.10 × 10^−3^
Spliceosome	61	4.17	2.20 × 10^−3^
Tyrosine metabolism	18	1.23	4.60 × 10^−2^

Percentage: the proportion of related proteins in relation to total number of proteins; *p*-value: probability of obtaining results.

## Supplementary Materials

The following are available online at https://www.mdpi.com/2304-8158/9/5/632/s1, Table S1: The list of total identified proteins of *Agaricus bisporus*. Table S2. The predicted transmembrane domains of the identified *Agaricus bisporus* proteins. Table S3. Table of functionally grouped proteins in *Agaricus bisporus*.
