# Supplementary material for: Increasing Coverage of Proteome Identification of the Fruiting Body of Agaricus bisporus by Shotgun Proteomics
Source: Foods. 2020 May 14;9(5):632. doi: 10.3390/foods9050632 (PMC7278689; doi:10.3390/foods9050632)
Supplement: Supplementary file 1 [file foods-09-00632-s001.zip › Supplementary Table 3.docx]

Supplementary File 3. Table of functionally grouped proteins in *Agaricus bisporus*

| Term | Number of related proteins | Percentage  (%) | P-Value |
| --- | --- | --- | --- |
| 2Fe-2S | 5 | 0.20 | 2.20E-02* |
| 4Fe-4S | 6 | 0.24 | 7.00E-02 |
| Actin-binding | 6 | 0.24 | 4.00E-02* |
| Amino-acid biosynthesis | 20 | 0.80 | 1.60E-06* |
| Aminoacyl-tRNA synthetase | 9 | 0.36 | 5.20E-03* |
| Aromatic amino acid biosynthesis | 6 | 0.24 | 1.90E-02* |
| ATP-binding | 165 | 6.64 | 1.90E-37* |
| Branched-chain amino acid biosynthesis | 7 | 0.28 | 7.00E-03* |
| Calcium | 5 | 0.20 | 9.50E-02 |
| Carbohydrate metabolism | 10 | 0.40 | 7.00E-02* |
| Chaperone | 16 | 0.64 | 4.40E-08* |
| Coiled coil | 318 | 12.80 | 3.30E-19* |
| Cytoplasm | 69 | 2.78 | 7.20E-19* |
| Cytoskeleton | 7 | 0.28 | 3.10E-02* |
| Decarboxylase | 6 | 0.24 | 1.90E-02* |
| DNA replication | 12 | 0.48 | 1.30E-02* |
| DNA-binding | 34 | 1.37 | 6.40E-03* |
| FAD | 20 | 0.80 | 2.60E-04* |
| Flavoprotein | 23 | 0.93 | 4.30E-05* |
| Glycolysis | 8 | 0.32 | 7.10E-04* |
| Golgi apparatus | 6 | 0.24 | 1.90E-02* |
| GTP-binding | 33 | 1.33 | 8.90E-07* |
| Helicase | 18 | 0.72 | 2.20E-04* |
| Hydrogen ion transport | 6 | 0.24 | 7.20E-03* |
| Hydrolase | 133 | 5.35 | 1.20E-09* |
| Initiation factor | 14 | 0.56 | 3.60E-05* |
| Ion transport | 8 | 0.32 | 8.80E-02 |
| Iron | 53 | 2.13 | 9.90E-04* |
| Iron-sulfur | 13 | 0.52 | 2.60E-04* |
| Isomerase | 22 | 0.89 | 5.90E-07* |
| Kinase | 53 | 2.13 | 3.60E-11* |
| Ligase | 23 | 0.93 | 5.50E-07* |
| Lipid biosynthesis | 9 | 0.36 | 3.00E-02* |
| Lipid metabolism | 12 | 0.48 | 1.30E-02* |
| Lyase | 27 | 1.09 | 7.60E-07* |
| Magnesium | 22 | 0.89 | 1.50E-05* |
| Manganese | 10 | 0.40 | 4.30E-03* |
| Metal-binding | 157 | 6.32 | 1.20E-10* |
| Microtubule | 7 | 0.28 | 8.10E-02 |
| Mitochondrion | 26 | 1.05 | 1.90E-02* |
| NAD | 24 | 0.97 | 2.80E-08* |
| NADP | 11 | 0.44 | 3.50E-03* |
| Nucleotide-binding | 204 | 8.21 | 2.80E-45* |
| Nucleotidyltransferase | 14 | 0.56 | 7.90E-03* |
| Nucleus | 74 | 2.98 | 1.80E-05* |
| One-carbon metabolism | 5 | 0.20 | 5.20E-02 |
| Oxidoreductase | 109 | 4.39 | 3.20E-13* |
| Protease | 40 | 1.61 | 2.30E-06* |
| Proteasome | 12 | 0.48 | 5.90E-06* |
| Protein biosynthesis | 28 | 1.13 | 2.70E-08* |
| Protein phosphatase | 5 | 0.20 | 5.20E-02 |
| Protein transport | 25 | 1.01 | 1.00E-06* |
| Pyridoxal phosphate | 18 | 0.72 | 1.20E-04* |
| Redox-active center | 4 | 0.16 | 6.60E-02 |
| Ribonucleoprotein | 44 | 1.77 | 1.90E-14* |
| Ribosomal protein | 42 | 1.69 | 5.10E-14* |
| RNA-binding | 17 | 0.68 | 7.90E-03* |
| Rotamase | 14 | 0.56 | 3.60E-05* |
| Serine/threonine-protein kinase | 31 | 1.25 | 3.10E-05* |
| SH3 domain | 13 | 0.52 | 5.90E-04* |
| Threonine protease | 12 | 0.48 | 5.90E-06* |
| Transcription | 21 | 0.85 | 9.30E-02 |
| Transferase | 124 | 4.99 | 5.50E-19* |
| Transit peptide | 12 | 0.48 | 2.90E-02* |
| Transport | 67 | 2.70 | 2.80E-06* |
| Tricarboxylic acid cycle | 5 | 0.20 | 5.20E-02 |
| Ubiquinone | 13 | 0.52 | 2.20E-03* |
| Ubl conjugation pathway | 19 | 0.76 | 4.40E-06* |
| WD repeat | 13 | 0.52 | 2.20E-02* |
|  |  |  |  |

Percentage: the proportion of related proteins in relation to total number of proteins; P-value: probability of obtaining results; *P<0.05
